# Adaptive colistin resistance dynamics in *mcr-9*-positive and -negative Enterobacterales following low-level colistin exposure

**DOI:** 10.1128/spectrum.02306-25

**Published:** 2026-05-29

**Authors:** Eva Smelikova, Marie Brajerova, Jan Tkadlec, Pavel Drevinek, Marcela Krutova

**Affiliations:** 1 Department of Medical Microbiology, Second Faculty of Medicine, Charles University and University Hospital Motol and Homolka 60568 , Prague, Czech Republic; 2 Department of Genetics and Microbiology, Faculty of Science, Charles University 112302 , Prague, Czech Republic; Centre de Biologie Integrative, Toulouse, France

**Keywords:** hospitalization, surveillance, prevalence, intestinal carriage, bioinformatics, MinION, plasmid-bound, hybrid assembly

## Abstract

The *mcr-9* gene has been reported across different sources and was recognized as a potential contributor to increased minimum inhibitory concentrations after colistin exposure. This study aimed to determine the prevalence of intestinal carriage of the *mcr-9* gene in hospitalized patients and characterize the genomic features of *mcr-9*-carrying Enterobacterales. From December 2019 to November 2020, rectal swabs were enriched and screened for the *mcr-9* gene using qPCR. PCR-positive broths were cultured on chromogenic agar, and *mcr-9*-positive isolates underwent whole-genome sequencing and antimicrobial susceptibility testing. Colistin susceptibility was reassessed after exposure to subinhibitory concentrations of colistin, including *mcr-9*-negative control isolates. Of 624 rectal swabs, 22 (3.5%) were *mcr-9*-positive, and 9 colistin-susceptible Enterobacterales were cultured. Following colistin exposure, resistance developed in 6/9 *mcr-9*-positive isolates and remained stable in 5/6 after 10 days of subcultivation on media without colistin; mutations in genes related to colistin resistance were identified. Colistin resistance also emerged in 4/7 *mcr-9*-negative isolates, with stable resistance observed in two. Exposure to low colistin concentrations can select for transient or stable resistance in Enterobacterales through diverse adaptive mechanisms. This phenomenon occurred in both *mcr-9*-positive and *mcr-9*-negative isolates and varied across strains, indicating that *mcr-9* presence alone does not predict adaptive outcomes. The heterogeneity in stability and reversibility of resistance suggests strain-specific genetic backgrounds and evolutionary fitness-related processes that warrant further investigation.

**IMPORTANCE:**

This study investigates the prevalence and genomic characteristics of *mcr-9*-carrying Enterobacterales in hospitalized patients, marking the first such screening in this population. Although the *mcr-9* gene is often associated with colistin-susceptible isolates, previous studies have suggested that exposure to colistin may be associated with increased minimum inhibitory concentrations in *mcr-9*-positive isolates. Our findings demonstrate that exposure to low colistin concentrations can select for colistin resistance in both *mcr-9*-positive and *mcr-9*-negative Enterobacterales, highlighting a heterogeneous and strain-dependent adaptive response. These resistance dynamics were primarily associated with mutations in regulatory systems such as PmrAB and PhoPQ and were not uniformly stable, with some isolates reverting to colistin susceptibility following growth in colistin-free media. This observation is clinically relevant, as subinhibitory colistin concentrations, commonly encountered due to unfavorable pharmacokinetics and dose-limiting nephrotoxicity, may inadvertently promote the emergence of adaptive resistance during treatment.

## INTRODUCTION

Colistin is a polypeptide antibiotic belonging to the polymyxin class that exhibits activity against multidrug-resistant gram-negative bacteria (1). Despite the availability of more effective and less toxic alternatives, colistin remains a last resort treatment due to its affordability (2). Until 2016, colistin resistance was rare and predominantly attributed to chromosomal mutations in two-component systems (1). However, in 2016, a plasmid-bound *mcr-1* gene, which mediates colistin resistance, was discovered (3). The MCR-1 phosphoethanolamine transferase, a membrane Zn^2+^ metalloenzyme, modifies lipid A, although the exact mechanism of its action remains unclear (4). The emergence of the *mcr-1* gene represents a significant threat to antibiotic efficacy, due to its potential for horizontal gene transfer (1). Since 2016, ten *mcr* variants have been identified (5–7), with global distribution across diverse reservoirs, including humans, animals, food, and the environment (8).

Isolates harboring molecular mechanisms of colistin resistance, whether mediated by chromosomal mutations or *mcr* genes, generally display minimum inhibitory concentration (MIC) values ranging from 4 to 16 mg/L. MIC values at the lower end of this range (4–8 mg/L) are particularly common among *mcr*-carrying isolates and may be difficult to detect using routine susceptibility testing methods (9, 10). Consequently, the use of inappropriate techniques, such as disk diffusion, may lead to the underdetection of resistance and false-susceptible interpretation (9–11).

Similarly, the culture of *mcr-9-* and *mcr-10*-positive isolates is challenging because these isolates are often colistin-susceptible (6, 7), limiting the effectiveness of selective culture methods (6, 7, 11, 12). Nonetheless, reduced susceptibility and even resistance to colistin in *mcr-9*-harboring isolates have been reported following exposure to colistin (6, 13). This study aimed to determine the prevalence of intestinal carriage of the *mcr-9* gene in hospitalized patients and characterize the corresponding bacterial isolates, with a particular emphasis on evaluating whether the *mcr-9* gene is associated with inducible colistin resistance after exposure to sub-inhibitory concentrations of colistin.

## MATERIALS AND METHODS

### Samples and *mcr-9*-positive isolate culture and identification

A prospective surveillance study was conducted at Motol University Hospital in Prague, Czech Republic, from December 2019 to November 2020.

Rectal swabs were collected from both adult and pediatric inpatients hospitalized at different wards. The swabs were enriched overnight at 37°C in 5 mL of *Enterobacteriaceae* enrichment broth (Mossel, Oxoid, UK). For direct detection of the *mcr-9* gene, 1 mL of enriched culture was used for DNA extraction using a DNeasy Blood & Tissue Kit (Qiagen, Germany). A qPCR assay with SYBR Green (Qiagen, Germany) with primers MCR9F (5′-CGGTACCGCTACCGCAATAT-3′) and MCR9R (5′-ATAACAGCGAGACACCGGTT-3′), along with a synthetic DNA *mcr-9*-positive control (GeneArt Strings, Thermo Fisher Scientific, Germany), was performed (6) using CFX96 instrument (Bio‐Rad Laboratories, USA), and the PCR protocol was as follows: 96°C/15 min; 35 cycles of 96°C/60s, 57°C/90s + plate read, and 72°C/60s; followed by 72°C/10 min. HRM analysis was performed at 70°C–95°C with 0:01 + plate read.

Since isolates harboring the *mcr-9* gene are frequently colistin-susceptible (6), *mcr-9*-positive broths were cultured overnight at 37°C on chromogenic agar without colistin (Brilliance UTI Clarity agar, Oxoid, UK). The grown colonies were further tested for the presence of the *mcr-9* gene as described above. The specificity of the *mcr-9*-positive PCR products was confirmed through Sanger sequencing. The species identification of the *mcr-9*-positive isolates was performed using Matrix-assisted laser desorption/ionization time-of-flight mass spectrometry (MALDI‐TOF/MS) Biotyper v3.1 (Bruker Daltonics, USA).

### Antimicrobial susceptibility testing

Antimicrobial susceptibility testing (AST) on 24 antimicrobials or colistin alone was performed on *mcr-9*-positive isolates using broth microdilution (BMD) (MIKRO-LA-TEST MIC, G minus I and II (*Enterobacteriaceae*), and/or MIC Colistin, Erba Mannheim, Germany) with Müeller-Hinton II cation-adjusted broth cultured overnight at 37°C. The results were interpreted using breakpoints according to the European Committee on Antimicrobial Susceptibility Testing (EUCAST) (14) or the Clinical Laboratory Standards Institute (CLSI) for tetracycline, netilmicin, and cefoperazone (15) (Table S1).

### Induction assay in the *mcr-9*-positive colistin-susceptible isolates and controls

The inducibility of colistin resistance was assessed in all *mcr-9*-positive colistin-susceptible isolates. The *mcr-9*-negative colistin-susceptible isolates of the same bacterial species were used as controls. Briefly, 100 µL of bacterial suspension of 0.5 McFarland density was inoculated into 5 mL of Luria-Bertani (LB) medium without colistin (growth control) and into four tubes containing colistin at concentrations of 0.064, 0.25, 0.5, and 1 mg/L, each in triplicate. The cultures were incubated overnight at 37°C under aerobic conditions on an orbital shaker (200 rpm, Biosan OS-10). Overnight cultures were centrifuged for 10 min at 2,460 × *g*, and the pellets were used to determine colistin MICs (MIC Colistin, Erba Mannheim, Germany). Colistin susceptibility testing was performed on the culture growing without colistin and on cultures at the highest colistin concentrations showing visible growth. These cultures were simultaneously inoculated onto chromogenic agar (Brilliance UTI Clarity agar, Oxoid, UK) with (3.5 mg/L) and without colistin. After overnight culture at 37°C, the bacterial species were confirmed using MALDI-TOF/MS, and the presence of the *mcr-9* gene was verified using qPCR.

To assess the stability of colistin resistance following exposure, isolates that developed colistin resistance, including both *mcr-9*-positive and *mcr-9*-negative isolates, were serially subcultured for 10 consecutive days on Brilliance UTI Clarity (Oxoid, UK) agar without colistin and incubated overnight at 37°C. Each day, grown colonies were identified by MALDI-TOF/MS, and colistin susceptibility was determined using BMD (MIC Colistin, Erba Mannheim, Germany) after overnight incubation at 37°C.

### EDTA-colistin broth disk elution testing

The EDTA-colistin broth disk elution (EDTA-CBDE) test was performed as described by Fenwick *et al*., with minor modifications (16) to investigate whether the chelation of metal cations, a requirement for MCR-1 function, also affects colistin resistance in inducible *mcr-9*-positive and *mcr-9*-negative colistin-resistant isolates.

Briefly, overnight cultures grown with a sub-inhibitory concentration of colistin were inoculated into 12 tubes containing Müller-Hinton broth (Oxoid, UK) and colistin disks (10 µg, Oxoid, UK) at final concentrations of 0, 1, 2, 4, 8, and 16 mg/L. Six tubes (set 1) contained EDTA (UltraPure 0.5M EDTA, pH 8.0, Invitrogen, USA, while the remaining six tubes served as EDTA-free controls (set 2). The cultures were incubated at 37°C without shaking. Differences of ≤1 dilution step were considered within normal methodological variation and interpreted as no effect of EDTA.

### Statistical analysis

The statistical significance between groups was assessed using Fisher’s two-sided exact test. A test value less than or equal to 0.05 was considered significant.

### Whole-genome sequence analysis

All *mcr-9*-positive isolates were characterized through short-read sequencing both before and after colistin exposure (if inducible). Additionally, one isolate was sequenced again after colistin susceptibility was restored following sub-culturing, along with the inducible *mcr-9-*negative control isolates.

DNA libraries for short-read sequencing were prepared using the Nextera XT DNA Library Preparation Kit (Illumina, USA) and outsourced for sequencing (Macrogen, South Korea).

All *mcr-9*-positive inducible isolates were sequenced using long-read sequencing prior to induction. DNA libraries were prepared using the Ligation Sequencing Kit, #SQK‐LSK109 (Oxford Nanopore Technologies, UK), or Native barcoding kit #SQK-NBD114.24, and sequencing was performed on a #FLO‐MIN106 or #FLO-MIN114 flow cell (Oxford Nanopore Technologies, UK). Long reads were assembled using Flye v2.9.1, polished with Medaka v1.7.2 and Polypolish v0.5.0.

The resulting fastq files and hybrid assemblies were analyzed using tools available at the Center for Genomic Epidemiology (https://www.genomicepidemiology.org/). KmerFinder 3.2 was used for verifying the bacterial species identified by MALDI-TOF/MS. Multilocus sequence types (MLST) were determined using MLST 2.0. Acquired antimicrobial resistance genes were identified using ResFinder 4.1, and PlasmidFinder 2.0 was used for plasmid classification. For the *Escherichia coli* isolate, serotype was determined by SerotypeFinder 2.0, phylogroup was identified by ClermonTyper, and virulence genes were analyzed using VirulenceFinder 2.0. Plasmid sequences containing *mcr-9* genes from hybrid assemblies were compared using the BLAST Ring Image Generator (BRIG). The surrounding genomic region of the *mcr-9* gene was compared using EasyFig v2.2.5.

To investigate the mechanism underlying colistin resistance inducibility and the restoration of colistin susceptibility, single-nucleotide polymorphism (SNP) analysis was performed using Snippy v4.6.0 (https://github.com/tseemann/snippy) on isolates before induction, after induction, and after susceptibility restoration. Complete genomes, obtained through hybrid assembly and annotated using RASTtk (https://rast.nmpdr.org/), served as reference genomes. Fastq reads from each experimental condition were then mapped and compared against these references.

## RESULTS

### Samples and *mcr-9*-positive isolate culture

Between December 2019 and November 2020, 624 rectal swabs from non-duplicated patients were investigated. The median and the average age of the patients were 9 and 23 years (range: 1 day to 93 years; SD: 27.2), respectively, and 55% of the patients were male. The *mcr-9* gene was detected in 22 samples (3.5%, 17 males and 5 females). Of these 22 *mcr-9*-positive samples, only 9 isolates (41%) were successfully cultured (*Enterobacter* spp., *n* = 6; *Citrobacter freundii*, *n* = 2; and *E. coli*, *n* = 1). None of the nine *mcr-9*-positive patients and seven *mcr-9*-negative colistin-susceptible controls received colistin.

Among the nine *mcr-9*-positive isolates, the most common resistance observed was to cefazolin (8/9, 89%), ampicillin (7/9, 78%), and ampicillin/sulbactam (5/9, 56%). All isolates were susceptible to colistin; the “skip well” phenomenon, indicating heteroresistance, was not observed. The summary of AST results is in Table S1.

### Emergence of colistin resistance after colistin exposure and its stability

Colistin resistance was observed in six *mcr-9*-positive isolates, including *C. freundii*, *E. coli,* and *Enterobacter roggenkampii* (67%), with MICs ≥ 16 in five isolates and 8 mg/L in one isolate after colistin exposure. Three *Enterobacter hormaechei* isolates (33%) remained susceptible, showing no increase in colistin MIC; the “skip well” phenomenon was not observed.

In *mcr-9*-negative controls, four isolates developed colistin-resistance after colistin exposure with MIC in the range 16 to ≥16 mg/L (4/7, 57%; *E. hormaechei, n* = 2; *E. roggenkampii*, *n* = 1; and *E. coli*, *n* = 1), while three isolates (*C. freundii, n* = 2 and *E. coli, n* = 1) remained colistin-susceptible; the “skip well” phenomenon was not observed. Induction assay results are summarized in Table S2.

There was no statistical difference in adaptation to colistin exposure between *mcr-9*-positive and *mcr-9*-negative isolates.

After 10 days of subculture, 70% (7/10) of isolates with acquired colistin resistance (five *mcr-9*-positive and two *mcr-9-*negative controls) retained their resistance. Three isolates reverted to colistin-susceptibility (one *mcr-9*-positive, P2211S, and two *mcr-9*-negative controls), with MICs ranging from 0.25 to 1 mg/L after 1–4 days of sub-cultivation.

### Genomic localization of the *mcr-9* gene

**Fig 1:**
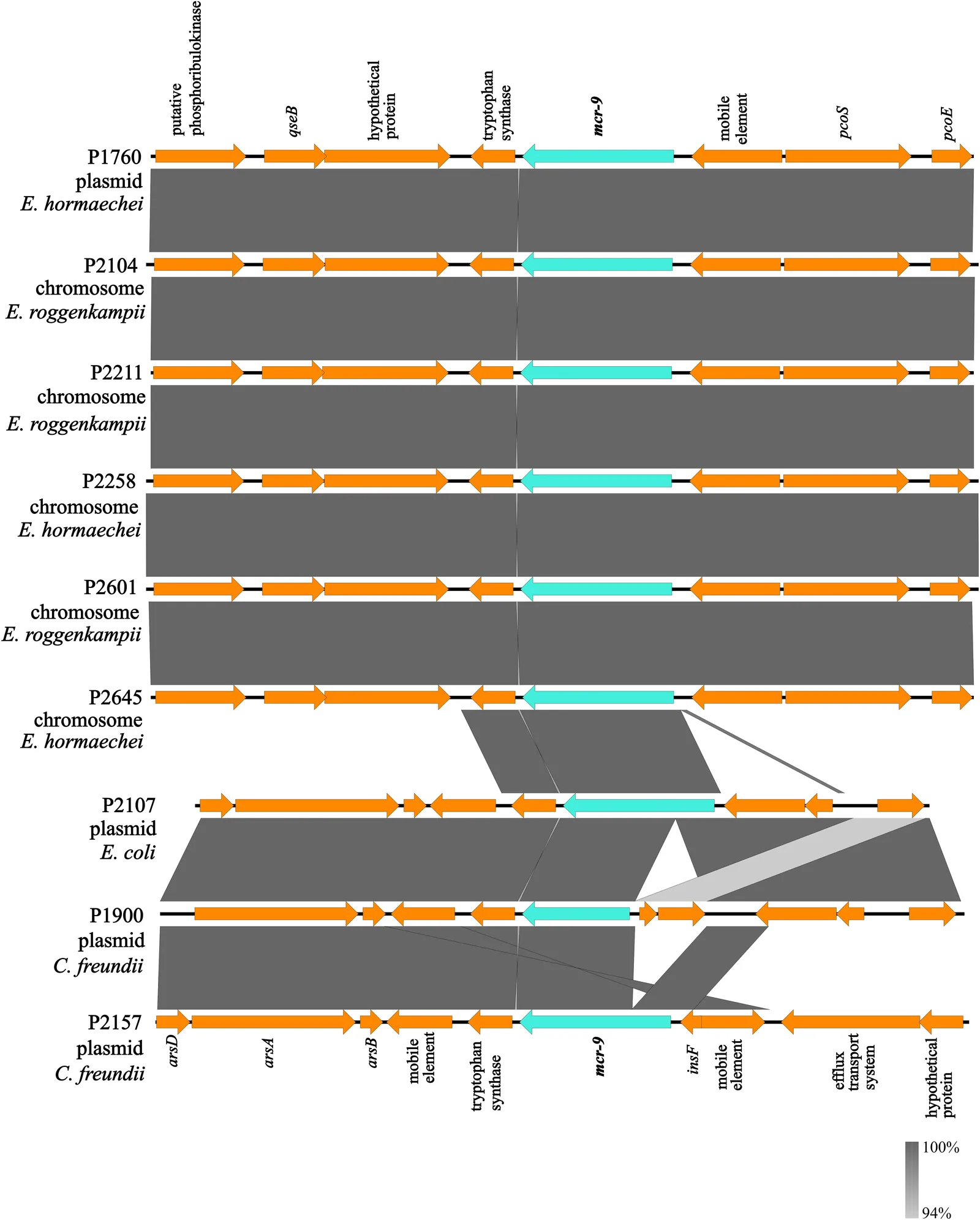
Localization of the *mcr-9* gene inside the plasmid or chromosome and its neighborhood. The ORF initially annotated as a hypothetical protein (adjacent to *qseB*) was confirmed to correspond to the *qseC* gene, based on its 100% identity and conserved synteny with the *mcr-9* locus in *E. coli* 68A (6). The *mcr-9* gene is highlighted in blue.

In all cases, the *mcr-9* gene was localized into arsenic, copper, and silver resistance (the MRG) gene cluster coding for heavy metal resistance, either at the beginning (pKPC-CAV1321, chromosome) or within the cluster (IncHI2 plasmid), regardless of colistin resistance inducibility (Fig. 1). The *mcr-9* genes identified in *E. hormaechei* and *E. roggenkampii* shared a consistent gene neighborhood, including the genes coding for the PcoSE, RcnAR proteins, and the *qseBC* genes. While these represent homologs of the canonical QseBC two-component system, they are identical to the *mcr-9*-associated regulatory locus (6). A second, chromosomal canonical QseBC system was also identified in all *mcr-9*-positive isolates, located distantly from the *mcr-9* gene.

In contrast, the *mcr-9* gene identified in *E. coli* and *C. freundii* on the pKPC-CAV1321 plasmid was surrounded by genes for ArsAB proteins and mobile genetic elements (Fig. 1). Notably, in one isolate (P1900), the *mcr-9* gene was split by two intervening genes encoding a hypothetical protein and a mobile element protein. Additionally, one *E. roggenkampii* isolate with a chromosomally located *mcr-9* gene also carried the *mcr-10* gene on an IncFIA(HI1) plasmid. The summary characteristics of the isolates are listed in Table 1.

**TABLE 1:** Characterization of colistin-susceptible Enterobacterales clinical isolates carrying the *mcr-9* gene and control isolates (without the *mcr-9* gene)

	Bacterial strain (KmerFinder)	MLST	Localization of the *mcr-9* gene	Detected plasmids	Inducibility	Mutations detected post-colistin exposure	EDTA reduction	ARGs^*f*^	Serotype	Phylogroup	Virulence genes
P1760	*E. hormaechei*	ST145	plasmid	IncHI2^*e*^, Col(pHAD28)	No	NA^*g*^	NA	*mcr-9*, *fosA*, *bla*_ACT-5_, *bla*_ACT-14_	NA	NA	NA
P2258	*E. hormaechei*	ST145	chromosome	Col(pHAD28), IncR	No	NA	NA	*mcr-9*, *fosA*, *bla*_ACT-5_, *bla*_ACT-14_	NA	NA	NA
P2645	*E. hormaechei*	ST278	chromosome^*a*^	IncHI2#, IncFIB(pECLA)	No	NA	NA	*mcr-9*, *bla*_ACT-5_, *bla*_ACT-14_	NA	NA	NA
1069	*E. hormaechei*	ST175	NA	Col(pHAD28), repA(dmsm701b), repB(R1701)	Yes	No	Yes	*fosA, bla_ACT-7_*	NA	NA	NA
1531	*E. hormaechei*	ST50	NA	Col(pHAD28)	Yes^*b*^	Yes^*c*^	Yes	*fosA, aph(6)-Id, bla_ACT-15_*	NA	NA	NA
P2104	*E. roggenkampii*	ST484	chromosome	IncFIB(pECLA)	Yes^*b*^	BasS/PmrB_P66L	No	*mcr-9*, *fosA*, *bla*_ACT-6_	NA	NA	NA
P2211	*E. roggenkampii*	ST1621	chromosome	IncFIA(HI1)^*d*^, Col(pHAD28), repB(R1701)	Yes	MgrB_K2fs	No	*mcr-9*, *mcr-10*, *bla*_ACT-10_	NA	NA	NA
P2601	*E. roggenkampii*	ST484	chromosome	IncFIB(pECLA)	Yes^*b*^	PhoQ_I178S	No	*mcr-9*, *fosA*, *bla*_ACT-6_	NA	NA	NA
S911B	*E. roggenkampii*	ST41	NA	Col(pHAD28), IncFIB(pECLA), IncFII(pECLA), repB(R1701)	Yes	No	No	*bla_MIR-3_, fosA*	NA	NA	NA
P1900	*C. freundii*	ST637	plasmid	pKPC-CAV1321^*e*^**,** IncFII(pRSB107),	Yes^*b*^	BasS/PmrB_D152A	Yes	*mcr-9*, *bla*_CMY-67_	NA	NA	NA
P2157	*C. freundii*	ST642	plasmid	pKPC-CAV1321^e^, IncFII	Yes^*b*^	BasS/PmrB_P66L_L17R	Yes	*mcr-9*, *bla*_TEM-1A_, *bla*_CMY-89_	NA	NA	NA
1076	*C. freundii*	NA	NA	NA	No	NA	NA	NA	NA	NA	NA
1405	*C. freundii*	NA	NA	NA	No	NA	NA	NA	NA	NA	NA
P2107	*E. coli*	ST1072/378	plasmid	pKPC-CAV1321^*e*^, IncX1	Yes^*b*^	BasR/PmrA_R81L	Yes	*mcr-9*	O4:H4	A	*clpK1, csgA, fdeC, fimH, gad, hha, hlyE, iss, lpfA, nlpl, terC, yehABCD*
1077	*E. coli*	ST131/43	NA	Col156, IncFIA, IncFIB(AP001918), IncFII(pRSB107), IncN4, IncX4	Yes^*b*^	BasS/PmrB_L197P	Yes	*aadA5, sul1, dfrA17, mph(A), tet(A), bla_CTX-M-27_*	O4:H4	B2	*aslA, chuA, csgA, fdeC, fimH, fyuA, gad, hha, iha, iss, iucC, iutA, kpsE, kpsMII, nlpl, ompT, papA, sat, senB, sitA, terC, usp, yehABCD, yfcV, yghJ*
1161	*E. coli*	NA	NA	NA	No	NA	NA	NA	NA	NA	NA

^*a*^ In P2645, the *mcr-9* gene is incorporated in the chromosome on the IncHI2. In P2258, P2211, P2601, and P2645, the *mcr-9 *gene is localized on the chromosome.

^*b*^ Isolates 1531, P2104, P2601, P1900, P2157, P2107, and 1077 remained resistant to colistin after 10 days of sub-cultivation.

^*c*^ Isolate 1531 had mixed nucleotide signals at positions 48–102 nt in the *mgrB* regulator sequence.

^*d*^ The plasmid carried the *mcr-10* gene.

^*e*^ Plasmids with the *mcr-9* gene.

^*f*^ ARGs – Antimicrobial resistance genes.

^*g*^ NA: not available.

### The mutation changes in the *mcr-9*-positive and *mcr-9*-negative isolates after colistin exposure

The SNP analysis of *mcr-9*-positive isolates, which acquired resistance after colistin exposure, before and after exposure, revealed missense and frameshift mutations in genes associated with colistin resistance. In six cases, the isolates before and after colistin exposure differ in only 0–2 SNPs. In P2211, an isolate with restored colistin susceptibility, a frameshift mutation (K2fs) in the MgrB regulator, initially detected after the development of colistin resistance, was lost after two rounds of subculture on antibiotic-free media, resulting in the restoration of colistin susceptibility. During subculture on colistin-free media, another mutation (R129Q) was detected in the PmrB regulator; however, the impact on colistin susceptibility is unclear.

In contrast, in *mcr-9*-negative isolates with newly acquired colistin resistance, only one isolate showed colistin-related substitution p.Leu197Pro in the BasS/PmrB, one showed mixed nucleotide signals at positions 48–102 nt in the *mgrB* regulator sequence, one had SNPs unrelated to colistin resistance, and one had shown no SNPs in comparison. In both isolate groups, SNPs not previously linked to colistin resistance were identified. Detailed SNP analysis results are provided in Table S3.

### EDTA-colistin broth disk elution testing

EDTA reduced colistin MICs in 7/10 *mcr-9*-positive and *mcr*-9-negative isolates with acquired colistin resistance (*C. freundii*, *E. coli,* and *E. hormaechei*), often with values below the breakpoint. The greatest reduction was observed in *E. hormaechei* (five-dilution reductions), where the MIC value was reduced from ≥16 mg/L to the susceptible category with 1 mg/L. *E. roggenkampii* showed a maximum MIC reduction of two dilutions, but the MIC value remains in the resistance range. The effect of EDTA appeared to be independent of mutations in colistin resistance-related proteins (Table 1; Table S4).

## DISCUSSION

The *mcr-9* gene was first described by Kieffer *et al*. (6) and has since been detected in human, animal, food, and environmental samples on six continents (6, 17). While the *mcr*-9 gene is associated with colistin-susceptible isolates (12, 18, 19), several studies suggested that *mcr-9*-positive strains may exhibit increased MIC after colistin exposure (6, 20). To our knowledge, this is the first study to evaluate the prevalence of intestinal carriage of Enterobacterales carrying the *mcr-9* gene in hospitalized patients. As a follow-up to our previous research on the prevalence of intestinal carriage of *mcr-1* to *8* genes, where *mcr-1* genes were detected at a low prevalence of 0.21% (10), this study found a prevalence of 3.5% for the *mcr-9* gene. However, only 9/22 (41%) *mcr-9-*positive samples yielded cultured isolates. The reason is that *mcr-9*-carrying isolates can show a multidrug-resistant phenotype, as well as susceptibility to a broad spectrum of antimicrobials, including colistin, and thus, designing selective culture media for screening is difficult (11, 21). The *mcr-9*-carrying strain isolation was also often complicated by the overgrowth of other bacteria, such as *Proteus* spp. Moreover, the growth of *Pseudomonas* spp. could cause *mcr-9*-positive enrichment, as *Pseudomonas* spp. were previously described as carriers of the *mcr-9* gene (22).

In this study, all *mcr-9*-positive isolates were colistin-susceptible and did not exhibit carbapenem resistance or extended-spectrum beta-lactamase (ESBL) production. However, the carbapenem-resistant colistin-susceptible *E. cloacae* harboring the *mcr-9* gene was identified from clinical samples in Japan (23). Additionally, five carbapenemase-producing colistin-susceptible Enterobacterales carrying *mcr-9* were detected in Czech hospitals in 2019 (12), in a study focused only on carbapenem resistance. The *mcr-9* gene was also identified in three ESBL-producing *E. hormaechei* isolates from a historical collection of clinical isolates in South Africa (24).

The inducibility of colistin resistance in *mcr-9*-harboring Enterobacterales has been reported previously (6). In our study, *mcr-9*-negative controls were included to determine whether the *mcr-9* gene is essential for this phenomenon. Interestingly, colistin resistance did not emerge in *mcr-9-*positive *E. hormaechei*, consistent with findings from the study of Li *et al*. (25). However, acquisition of colistin resistance was observed in *E. coli* and *E. cloacae* isolates, as reported in previous studies (6, 20). Furthermore, *mcr-9*-positive *C. freundii* isolates exhibited an increase in MIC following growth in the presence of colistin, whereas *mcr-9*-negative isolates showed no change. Conversely, the development of colistin resistance in *mcr-9*-negative *E. hormaechei* suggests that chromosomal mutations, rather than the presence of the *mcr-9* gene, were the primary drivers of increased colistin MIC. This interpretation is further supported by gene cloning experiments demonstrating that *mcr-9* and *mcr-10* alone do not confer colistin resistance (26). Collectively, these findings align with evidence that some antibiotics, including fluoroquinolones and beta-lactams, increase bacterial mutation rates, potentially leading to resistance (27).

In this study, the *mcr-9* gene was localized on chromosome or plasmid replicons (IncHI2/pKPC-CAV1321) in 56% and 44% of isolates, respectively. The neighborhood of the *mcr-9* gene in the isolates from our study could be divided into two groups, as it was localized either at the beginning (pKPC-CAV1321) or inside the MRG cluster (IncHI2, chromosome). The *mcr-9* gene, localized at the beginning of the MRG cluster, was surrounded by heterogeneous mobile genetic elements that differed among isolates, indicating dynamic transmission of the gene.

The EDTA-colistin broth disk elution test has been used to differentiate plasmid-mediated colistin resistance (e.g. *mcr-1*-gene mediated), with a substantial MIC reduction, from resistance mediated by chromosomal mutations, which show minimal or no decrease in MIC (28). In this study, EDTA reduced the colistin MIC for most *mcr-9*-positive and *mcr-9*-negative isolates with acquired colistin resistance. On the other hand, EDTA does not have an effect on most *E. roggenkampii* isolates, where the *mcr-9* gene was localized inside the MRG cluster of the chromosome or the *mcr-9* was not present at all, which may indicate distinct responses to environmental stimuli affecting colistin susceptibility independent of *mcr-9*. Moreover, as EDTA does not specifically target colistin resistance genes but instead alters ionic availability in the surrounding environment, further transcriptomic and/or proteomic analyses are required to elucidate the mechanisms underlying the emergence of *mcr*-independent colistin resistance.

Our data resulting from comparative genomic analyses and phenotypic observations of colistin susceptibility suggest that colistin resistance in *mcr*-9-positive isolates is primarily driven by mutations in genes involved in lipopolysaccharide biosynthesis, particularly the PmrAB and PhoPQ two-component systems, independent of the *mcr-9* gene. An amino acid substitution in the PmrA protein at position 81 appears to be critical for colistin resistance, as several studies, including ours, have reported this mutation at the same locus (29, 30). In some species, mutations in these two-component systems are linked with their overexpression, conferring colistin resistance (31). However, these mutations may also alter protein function without overexpression, as observed previously in *E. cloacae* (32).

The variability in response to colistin exposure among *E. hormaechei* isolates may depend on phylogenetic clusters, as previously suggested (33). In our study, all three *mcr-9*-positive *E. hormaechei* isolates, in which resistance was not observed after colistin exposure, belonged to ST145, with the *mcr-9* gene localized on either the plasmid or the chromosome. This aligns with findings from a study published by Li et al., who reported unchanged *mcr-9* gene expression levels and the plasmid replication protein *repA* after the cultivation of *E. hormaechei* isolates with subinhibitory concentrations of colistin. According to their study, the lack of a *qseBC* two-component system is responsible for the non-inducibility of colistin resistance (25). However, in our study, the *qseBC* genes were detected regardless of the phenotypic response to colistin exposure.

Colistin resistance stability testing revealed that *E. coli* and *C. freundii* remained resistant after 10 days of subcultivation; however, some isolates of *E. hormaeche*i and *E. roggenkampii* became susceptible, regardless of the presence of the *mcr-9* gene. The *mcr-9-*positive *E. roggenkampii* isolate, sequenced after two subcultures, during which colistin susceptibility was restored, lacked the frameshift mutation in MgrB (K2fs) that had appeared following colistin exposure. A frameshift mutation in the *mgrB* gene causes premature termination of translation, loss of protein function, and colistin resistance (34). Also, a new mutation in PmrB (R129Q) was identified; however, further study is needed to elucidate the impact of this mutation on colistin susceptibility.

The limitation of this study is its design, which is based on comparative genomic analyses and phenotypic observations of colistin susceptibility in a limited number of isolates. To generalize these findings, future work should include a broader set of isolates, both carrying and lacking the *mcr-9* gene and a proteomic or transcriptomic approach to examine how the expression and activity of multiple genes involved in colistin resistance are affected.

### Conclusion

Exposure to low colistin concentrations can select for transient or stable resistance in Enterobacterales through diverse adaptive mechanisms. This phenomenon occurred in both *mcr-9*-positive and *mcr-9*-negative isolates and varied across strains, indicating that *mcr-9* presence alone does not predict adaptive outcomes. The heterogeneity in stability and reversibility of resistance suggests strain-specific genetic backgrounds and evolutionary fitness-related processes that warrant further investigation.

## Data Availability

All raw sequences of the isolates before and after colistin induction (if inducible) and hybrid assembly of isolates which were inducible have been submitted to the NCBI Sequence Read Archive (SRA) database with a BioProject ID: PRJNA1237247. Bacterial strains used in our study are stored at −80°C and are available upon request.

## SUPPLEMENTAL MATERIAL

Supplemental tables spectrum.02306-25-s0001.xlsx Tables S1 to S4.

ASM does not own the copyrights to Supplemental Material that may be linked to, or accessed through, an article. The authors have granted ASM a non-exclusive, world-wide license to publish the Supplemental Material files. Please contact the corresponding author directly for reuse.
